# The Importance of pH in Regulating the Function of the *Fasciola hepatica* Cathepsin L1 Cysteine Protease

**DOI:** 10.1371/journal.pntd.0000369

**Published:** 2009-01-27

**Authors:** Jonathan Lowther, Mark W. Robinson, Sheila M. Donnelly, Weibo Xu, Colin M. Stack, Jacqueline M. Matthews, John P. Dalton

**Affiliations:** 1 Institute for the Biotechnology of Infectious Diseases (IBID), University of Technology Sydney (UTS), Ultimo, Sydney, New South Wales, Australia; 2 School of Molecular and Microbial Biosciences, University of Sydney, New South Wales, Australia; Queensland Institute of Medical Research, Australia

## Abstract

The helminth parasite *Fasciola hepatica* secretes cathepsin L cysteine proteases to invade its host, migrate through tissues and digest haemoglobin, its main source of amino acids. Here we investigated the importance of pH in regulating the activity and functions of the major cathepsin L protease *Fhe*CL1. The slightly acidic pH of the parasite gut facilitates the auto-catalytic activation of *Fhe*CL1 from its inactive pro*Fhe*CL1 zymogen; this process was ∼40-fold faster at pH 4.5 than at pH 7.0. Active mature *Fhe*CL1 is very stable at acidic and neutral conditions (the enzyme retained ∼45% activity when incubated at 37°C and pH 4.5 for 10 days) and displayed a broad pH range for activity peptide substrates and the protein ovalbumin, peaking between pH 5.5 and pH 7.0. This pH profile likely reflects the need for *Fhe*CL1 to function both in the parasite gut and in the host tissues. *Fhe*CL1, however, could not cleave its natural substrate Hb in the pH range pH 5.5 and pH 7.0; digestion occurred only at pH≤4.5, which coincided with pH-induced dissociation of the Hb tetramer. Our studies indicate that the acidic pH of the parasite relaxes the Hb structure, making it susceptible to proteolysis by *Fhe*CL1. This process is enhanced by glutathione (GSH), the main reducing agent contained in red blood cells. Using mass spectrometry, we show that *Fhe*CL1 can degrade Hb to small peptides, predominantly of 4–14 residues, but cannot release free amino acids. Therefore, we suggest that Hb degradation is not completed in the gut lumen but that the resulting peptides are absorbed by the gut epithelial cells for further processing by intracellular di- and amino-peptidases to free amino acids that are distributed through the parasite tissue for protein anabolism.

## Author Summary

*Fasciola hepatica* is a helminth parasite that causes liver fluke disease (fasciolosis) in domestic animals (sheep and cattle) and humans worldwide. Cathepsin L cysteine proteases (*Fhe*CL) are secreted by the parasite to invade its host, migrate through tissues and to degrade host haemoglobin (Hb), a major source of nutrient to the parasite. *Fhe*CL1 is a very stable protease and active over a broad pH range (3.0–9.0), making it very suitable for functions both inside and outside the parasite. The slightly acidic pH of the parasite gut not only regulates the autocatalytic activation of the pro*Fhe*CL1 zymogen to an active *Fhe*CL1 protease but also induces relaxation of the Hb structure, making it more susceptible to proteolysis. The action of *Fhe*CL1, which is enhanced by glutathione (GSH), the major reducing agent found in red blood cells, degrades Hb to small peptides (predominantly 4–14 residues) that can be absorbed by the gut epithelial cells. Further processing within these cells by exopeptidases provides the necessary amino acids required for protein anabolism by the parasite.

## Introduction

Fasciolosis is a disease caused by helminths of the genus *Fasciola*. *F. hepatica* is found in temperate climates whereas *F. gigantica* is predominant in tropical regions. However, the distribution of the two species overlap in the Asia-Pacific region where hybrid forms have been isolated [1],[2]. Fasciolosis is of major global economic importance as it infects primary production livestock of humans, especially sheep, cattle and water buffalo. Moreover, epidemiological surveys carried out over the last 15 years have uncovered fasciolosis as a significant human zoonosis. To-date, high prevalence of human infection has been reported in South America (Ecuador, Peru and Bolivia), Vietnam, Thailand, Egypt and Iran [2],[3]. Animals and humans become infected by ingesting vegetation contaminated with infective larvae which emerge from cysts and migrate through the intestinal wall and liver tissue causing extensive tissue damage and haemorrhaging as they burrow and feed. The parasites then enter the bile ducts where they mature and produce eggs that are carried into the environment with the bile juices [1].

The success of *F. hepatica* as a parasite is related its ability to infect and complete its lifecycle in wide range of mammalian hosts. Besides domestic ruminants and humans these include a large number of relevant reservoir hosts, such as deer, rabbits, hares, rats and mice [2]. Over the last few centuries European colonisation accelerated the distribution of the disease by introducing infected animals into many countries [2],[3]. Most remarkably, within this relatively brief time period the parasite has adapted to local host species such as camelids in Africa, llamas and alpaca in South America and kangaroo in Australia [2].

*Fasciola* parasites infect and survive in their hosts by secreting cathepsin cysteine proteases. RNAi-mediated knock-down of cysteine protease activity of infective larvae was shown to prevent their ability to migrate through the host intestinal wall [4]. Also, blocking the function of these enzymes using anti-cysteine protease inhibitors or by vaccination with purified enzymes protects animals from infection [5],[6]. The primary function of the *F. hepatica* cathepsin L proteases is in the digestion of host haemglobin (Hb), the main source of nutrient for the parasite. This takes place within the lumen of the parasite gut, which is believed to be slightly acidic at around pH 5.5 [7],[8]. Adult parasites draw blood with a muscular pharynx through punctures they make in the wall of the bile duct and use it to supply the amino acids needed for the massive production of eggs [7]. Due to the blind-ended nature of the adult parasite gut it must be emptied regularly by regurgitation (approximately every three hours) and refilled with fresh blood [9]. This process is also important for the extrusion of the cathepsin L proteases into the host tissues where they are involved in additional pivotal functions to parasitism including penetration of the host's tissues, cleavage of host immunoglobulins and suppression of immune cell proliferation [3],[6]. These extracorporeal functions of the parasite enzymes are performed, therefore, in an environment of neutral physiological pH.

The functions of proteases are not only related to their physico-biochemical properties but also to their cellular/tissue location and physiological environment (particularly pH). Since *F. hepatica* cathepsin L proteases are required to function both inside and outside the parasite we considered it important to investigate the regulatory influence of pH on the autocatalytic processing and activation of the inactive zymogen, and on the structural stability and hydrolytic activity of the major secreted enzyme cathepsin L1 (*Fhe*CL1). We found that this enzyme was most rapidly activated at low pH but, once activated, was stable and functional over a broad pH range with an optimal hydrolytic activity at pH 6.2. While *Fhe*CL1 readily cleaved peptide and protein (ovalbumin) substrates at neutral pH, Hb was resistant to cleavage at this pH. The degradation of Hb required acid-induced structural changes that made it susceptible to *Fhe*CL1 cleavage. Degradation was enhanced by the presence of small thiol agents, such as glutathione and cysteine, which activate *Fhe*CL1 and are present in physiologically-relevant concentrations in red blood cells and plasma. Our experiments suggest that Hb is digested by the parasite in a microenvironment, likely between the lamellae of the gut epithelial cells, at a pH of approximately 4.5. Under low pH and reducing conditions *Fhe*CL1 is capable of generating small peptides but not free amino acids. We propose that these peptides are absorbed by the gut epithelial cells of the parasite where further processing takes place by intracellular dipeptidases [10] and aminopeptidases [11] to release amino acids that are distributed to the parasites tissues and used for protein anabolism.

## Materials and Methods

### Materials

Z-Phe-Arg-NHMec was obtained from Bachem (St. Helens, UK). E-64, DTT, l-cysteine, GSH (reduced glutathione), EDTA and ovalbumin were obtained from Sigma-Aldrich (Sydney, Australia). Prestained molecular mass markers were obtained from Invitrogen (Victoria, Australia).

### Activation of pro*Fhe*CL1 to mature *Fhe*CL1

Expression, production and purification of recombinant wild-type pro*Fhe*CL1 and variant pro*Fhe*CL1Gly^25^ (procathepsin L) in the yeast *Pichia pastoris* have been described elsewhere [12],[13]. The variant pro*Fhe*CL1Gly^25^ is an inactive zymogen since the active site Cys was replaced by a Gly. Auto-activation of the active wildtype pro*Fhe*CL1 was carried out by incubating 0.2 mg/ml enzyme at 37°C in 100 mM sodium acetate buffer, pH 4.5, containing 1 mM DTT and 1 mM EDTA. Aliquots (15 µl) were removed at time intervals and added to tubes containing 1 µl of 1 mM E-64 to stop the reaction. Proteolytic cleavage of the prosegment was visualised by 15% SDS-PAGE.

Auto-activation was also monitored in the presence of the fluorogenic substrate Z-Phe-ArgNHMec by measuring the release of fluorescence over time using a KC4 Bio-Tek micro-plate reader in 96-well fluorescent plates. pro*Fhe*CL1 (5 nM) was incubated in 100 mM buffer pH 4.0–pH 7.0 in the presence of 2 µM Z-Phe-Arg-NHMec. Final linear rates of substrate hydrolysis at each pH were measured with *Fhe*CL1 after auto-catalysis was completed.

### Fluorescence assays for activity of mature *Fhe*CL1

Fluorescence assays measuring activity of mature *Fhe*CL1 was carried out in 96-well plates using a KC4 Synergy HT micro-plate reader (Bio-Tek Instutments Inc., Vermont, USA). Assays were carried out with a final substrate concentration of 0.5 µM in a volume of 200 µl. When [S]<K_M_ the initial rate is proportional to k_cat_/K_m_. Assays contained 0.14 nM cathepsin L1 in the following buffers: 100 mM formate (pH 3.24–4.0), 100 mM sodium acetate (pH 4.0–5.5), 100 mM sodium phosphate (pH 5.5–8.0), and 100 mM sodium borate (pH 8.0–10.0). The assay also contained final concentrations of 1 mM DTT and 1.0 mM EDTA. The data were fitted to the equation:
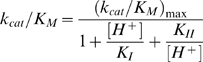



Stability of *Fhe*CL1 was investigated by incubating 0.1 mg/ml enzyme in 100 mM buffer (pH 2.5–pH 9.0) at 37°C. Enzyme activity towards 5 µM Z-Phe-Arg-NHMec in 100 mM sodium acetate buffer, pH 5.5 and containing 1 mM DTT was measured at time intervals over a 10-day period.

### Preparation of red blood cell lysates

Human red blood cells were washed three times by resuspending 0.25 ml of whole blood in 5 ml PBS and centrifugation at 5000 rpm. The supernatant with the buffy coat was removed each time. After the final wash, the cells were lysed to release haemoglobin (Hb) by adding 1 ml ice-cold distilled H_2_O for 10 min and then the suspension was centrifuged at 15000 rpm to remove insoluble material [14]. To remove any free amino acids or low molecular mass material Hb was dialysed twice against 1.5 L phosphate-buffered saline (PBS), pH 7.3, for 3 h using a dialysis membrane with a 3000 Da molecular mass cut-off (Sigma Chemical Co., Sydney, Australia). Hb was quantified using an extinction coefficient of 125 000 M^−1^ cm^−1^ at 414 nm [15] and was in good agreement with the total protein in lysates measured by the Lowry method [16] using BSA as standard.

### Spectrophotometric measurement of Hb denaturation

Spectrophotometry was carried out in in 96-well plates in a KC4 Synergy HT micro-plate reader. Hb was diluted to a final concentration of 5 µM into 100 mM buffer and denaturation was recorded for one hour by monitoring the decrease in absorbance at 414 nm [15]. Buffers used were 100 mM sodium acetate buffer, pH 3.5–pH 5.5 and 100 mM sodium phosphate buffer, pH 6.0–pH 7.0. Absorption spectra were recorded after one hour for each sample from 600 nm–300 nm. Hb denaturation was also monitored for 90 minutes at pH 4.5 and pH 7.0 in the presence of 1 mM GSH, with or without *Fhe*CL1.

### Circular dichroism (CD) of pro*Fhe*CL1Gly^25^

Stock *Fhe*proCL1Gly^25^ in PBS was dialysed into 50 mM sodium phosphate buffer, pH 7.5 or 50 mM sodium acetate buffer, pH 4.0, to remove any NaCl that could interfere with the CD spectrum. CD spectra of 5.3 µM pro*Fhe*CL1Gly^25^ (∼0.2 mg/ml) were recorded over the wavelength range 195–250 nm, in steps of 0.5 nm and speed of 20 nm/min using a Jasco720 spectropolarimeter in quartz cuvettes with a 0.1 cm pathlength. Spectra were the average of three scans and were buffer baseline corrected.

### SDS-PAGE analysis of Hb and ovalbumin digestion by *Fhe*CL1

Hb (1.8 nmoles) and ovalbumin (1.2 nmoles) were incubated with *Fhe*CL1 (0.18 nmoles) in 0.1 M buffers, pH 3.5–8.0 and containing 1 mM DTT. Control experiments contained no enzyme. The buffers used were 100 mM sodium acetate (pH 3.5–5.5) and 100 mM sodium phosphate (pH 5.5–8.0). The reactions were stopped after 30 min by adding 1 µl 1 mM E-64 to the tube and aliquots were analysed by 15% SDS-PAGE under reducing conditions. Gels were stained with a 0.1% w/v solution of Coomassie Brilliant Blue R-250 in 40% methanol/10% acetic acid [17].

### Analysis of Hb proteolysis by mass spectrometry

Hb (1.8 nmoles) was digested with purified recombinant *Fhe*CL1 (0.9 nmoles) in 0.1 M sodium acetate buffer (pH 4.0) containing 1 mM GSH and 1 mM EDTA for 0, 10, 20, 30, 45, 60, 75, 90, 120 and 180 minutes at 37°C. 10 µl aliquots of the digests were analysed using NuPage Novex 4–12% Bis-Tris gels (Invitrogen) according to the manufacturer's instructions. Gels were stained with Colloidal Coomassie Blue G250 (Sigma).

Hb digests were spun at 13,000 rpm for 15 min to remove particulates and were concentrated to a final volume of 15 µl using a Concentrator 5301 (Eppendorf). Peptides were analysed by nanoLC-ESI-MS/MS using a Tempo nanoLC system (Applied Biosystems) with a C18 column (Vydac) coupled to a QSTAR Elite QqTOF mass spectrometer running in IDA mode (Applied Biosystems). Peak list files generated by the Protein Pilot v1.0 software (Applied Biosystems) were exported to local MASCOT (Matrix Science) and PEAKs (Bioinformatics Solutions Inc.) search engines for protein database searching. MS/MS data was used to search 3239079 entries in the MSDB (20060809) database using MASCOT whereas PEAKs software was used to search a custom-made database containing only human Hb-alpha and Hb-beta sequences. The peptide mass tolerance was set at 0.1 Da, oxidation of methionine residues was set as a variable protein modification and the “no enzyme” function was selected. For MASCOT searches, matches with a MOWSE score >70 were considered to be significant [18],[19] and matched peptides achieving a score >60% were accepted during PEAKs searches. The matching peptides were then mapped onto the primary amino acid sequences of human Hb-alpha and Hb-beta to identify *Fhe*CL1 cleavages sites.

For matrix-assisted laser desorption ionisation time-of-flight mass spectrometry (MALDI-TOF MS) the Hb digest was desalted and concentrated by zip-tip (Millipore Perfect Pure C18) and spotted using 1 µL matrix (α-cyano-4-hydroxycinnamic acid, 4 mg/mL in 70% v/v acetonitrile, 0.06% v/v TFA, 1 mM ammonium citrate) onto a target plate, and allowed to air dry (Australian Proteome Analysis Facility, Macquarie University, Sydney.). The sample was then analysed using a 4700 Proteomics System TOF mass spectrometer (Applied Biosystems, USA) operated in reflectron mode in the mass range of 100 *m/z* to 400 *m/z*. Spectra were analysed manually and externally calibrated using ACTH (fragment 18–37), neurotensin, angiotensin I, bradykinin to give a mass accuracy 50 ppm or less.

## Results

### Autocatalytic activation of the pro*Fhe*CL1 zymogen to an active mature enzyme occurs most quickly at low pH

**Figure 1:**
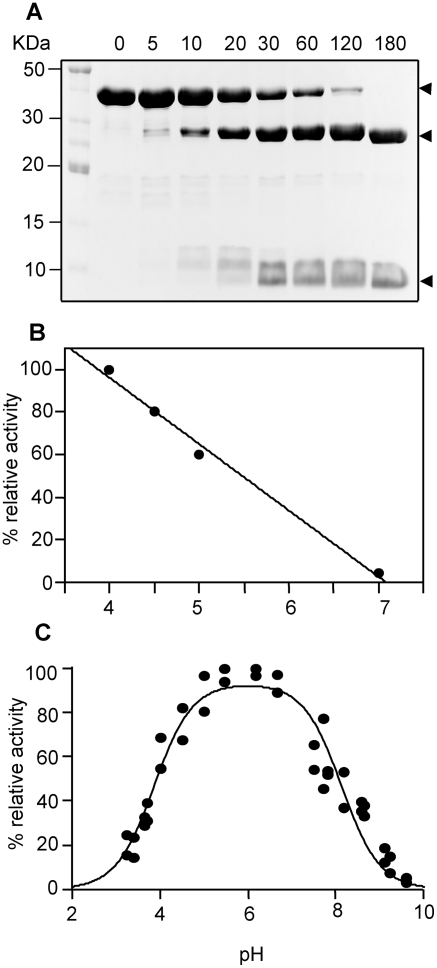
Influence of pH on zymogen pro*Fhe*CL1 autocatalytic activation and activity of mature *Fhe*CL1. (A) Analysis by SDS-PAGE of the activation of 0.2 mg/ml *Fhe*proCL1 to mature *Fhe*CL1 in 0.1 M sodium acetate buffer, pH 4.5. The zymogen, mature enzyme and degraded prosegment are indicated by arrowheads. (B) Kinetic study of the activation of 5 nM *Fhe*proCL1 between pH 4.0 and pH 7.0 in the presence of 2 µM Z-Phe-Arg-NHMec. (C) Relative k_cat_/K_m_ values for the hydrolysis of 0.5 µM Z-Phe-ArgN-Mec by 0.14 nM mature *Fhe*CL1 at 37°C.

The zymogen of the *F. hepatica* cathepsin L1, pro*Fhe*CL1 (Mr ∼38 kDa) is auto-catalytically processed at pH 4.5 by inter-molecular cleavage and removal of the prosegment to release a fully mature and active enzyme (Mr ∼25 kDa) (Figure 1A). Analysis of the *in vitro* auto-activation process by 4–20% SDS-PAGE shows that a band corresponding to the processed ∼25 kDa mature enzyme is observed within 5 minutes and that full removal of the prosegment from the zymogen occurs between two and three hours. Peptides representing products of the cleaved prosegment are observed below the 10 kDa molecular size marker (Figure 1A).

The rate of formation of an active mature enzyme from the inactive zymogen (5 nM) was monitored between pH 4.0–7.0 by performing the autocatalytic reaction in the presence of the fluorogenic substrate Z-Phe-Arg-NHMec and calculating the rate of hydrolysis (Figure 1B). The rate of hydrolysis of Z-Phe-Arg-NHMec, and hence the rate of activation from pro*Fhe*CL1 to *Fhe*CL1, was linear over this pH range; however, hydrolysis at pH 4.0 was ∼40-fold greater than at pH 7.0 indicating that autocatalytic activation occurs much more rapidly in an acidic environment (Figure 1B).

### Mature *Fhe*CL1 exhibits optimal activity at pH 6.2

The relationship between the activity of the fully processed mature *Fhe*CL1 and pH was examined by determining the k_cat_/K_m_ against Z-Phe-Arg-NHMec at various pH values in the range 2–10. The results show that the enzyme has the capacity to cleave substrates over a wide pH range (pH 3.0–9.0). Maximal activity was observed between pH 5.5–7.0 with a peak at pH 6.2 (Figure 1C).

### The zymogen pro*Fhe*CL1 and mature *Fhe*CL1 are stable over a wide pH range

**Figure 2:**
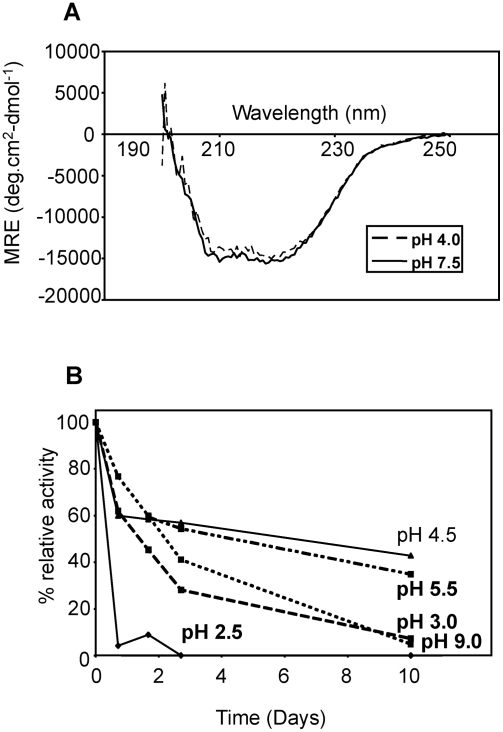
Stability of the zymogen pro*Fhe*CL1Gly^25^ and mature *Fhe*CL1 at various pH values. (A) Far-UV CD spectra of 5.3 µM *Fhe*proCL1Gly^25^ in 50 mM sodium acetate buffer, pH 4.0 and in 50 mM sodium phosphate buffer, pH 7.5. (B) Enzymatic stability of 6.0 µM mature *Fhe*CL1 at 37°C and in 0.1 M buffers over the pH range 2.5–9.0. Enzyme activity was monitored at various time-points by diluting aliquots of the reactions into 0.1 M sodium acetate buffer, pH 5.5, containing 3 mM DTT before addition of 5 µM Z-Phe-Arg-NMec.

We recently described the production of a catalytically inactive pro*Fhe*CL1 zymogen by replacing the active site Cys^25^ with a Gly [12],[13]. This variant, pro*Fhe*CL1Gly^25^, was correctly folded but inactive and, therefore, unable to autocatalytically activate even at low pH. In the present study, we used this pro*Fhe*CL1Gly^25^ variant to examine the stability of the zymogen at various pH values by subjecting it to analysis by circular dichroism (CD) in various solutions buffered in the pH range 4.0–7.5 (Figure 2A). No significant difference in the far-UV CD spectra of pro*Fhe*CL1 was observed showing that no conformational shifts occur in the secondary structure over this pH range. These data indicate that pro*Fhe*CL1 would remain stable during the auto-catalytic activation process to *Fhe*CL1, even at pH 4.0.

To investigate the susceptibility of the mature activated enzyme to pH denaturation, mature *Fhe*CL1 was incubated for at various time-periods at 37°C in buffers over the pH range 2.5–9.0 and then assayed for activity towards Z-Phe-Arg-NHMec in the presence of 1 mM DTT (Figure 2B). The enzyme exhibited optimal stability at pH 4.5; even following a 10-day incubation period the enzyme retained ∼45% activity at pH 4.5 and ∼5% activity at pH 3.0 demonstrating that *Fhe*CL1 is very stable in a moderately acidic environment. When incubated at pH 2.5, enzyme activity was not completely lost until day three.

### The hydrolytic activity of *Fhe*CL1 is enhanced by glutathione and cysteine at physiologically-relevant concentrations

**Figure 3:**
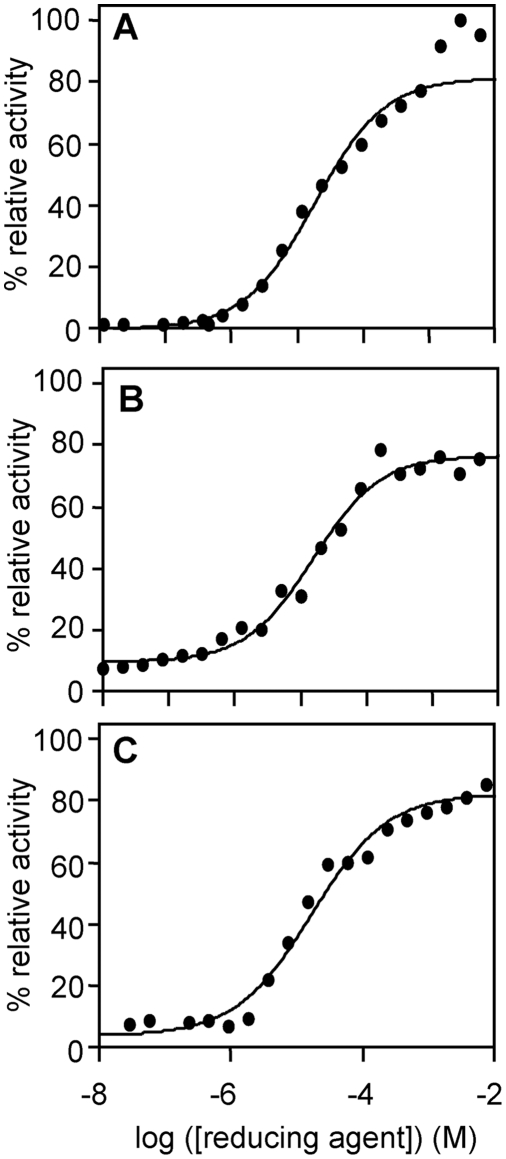
Effect of small molecular thiols on the activity of *Fhe*CL1. *Fhe*CL1 was incubated with (A) DTT, (B) GSH and (C) l-cysteine before adding the fluorogenic substrate Z-Phe-Arg-NHMec. Final assays contained 4 nM enzyme, (10 nM–10 mM) reducing agent and 5 µM substrate in 0.1 M sodium acetate buffer, pH 4.5, with 1 mM EDTA.

The activity of cysteine proteases is enhanced in the presence of small thiol molecules that reduce the active site cysteine. Dithiothreitol (DTT) is typically included in reactions carried out in the laboratory, but since this is not a physiological-relevant thiol we investigated whether reduced glutathione (GSH) and cysteine could activate the mature *Fhe*CL1 at concentrations found in blood (GSH is found predominantly in red blood cells at concentrations of approximately 1.2 mM, while cysteine is found in plasma at 0.23 mM, [20]). To do this *Fhe*CL1 was incubated for 5 minutes at pH 4.5 in a range of concentrations of dithiothreitol (DTT), GSH and L-cysteine. Substrate (Z-Phe-Arg-NHMec) was then added and endopeptidase activity determined by monitoring release of -NHMec with time. We found that in the presence of DTT, GSH and L-cysteine *Fhe*CL1 exhibited similar activation curves with maximal enzyme activity observed in the presence of each reducing agent at a concentration of ∼0.1 to 1.0 mM (Figure 3).

### *Fhe*CL1 requires acid pH to degrade Hb but can degrade ovalbumin from pH 3.5 to pH 8.0

**Figure 4:**
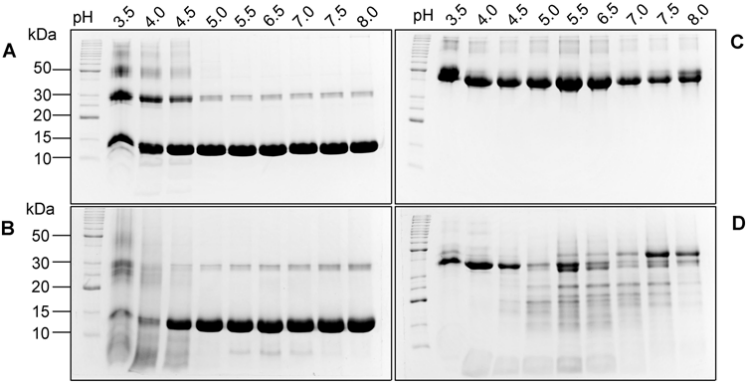
pH dependency of *Fhe*CL1 hydrolytic activity against protein substrates Hb and ovalbumin. (A) Hb incubated alone in solutions buffered in the ranges pH 3.5–pH 8.0; (B) Hb incubated with *Fhe*CL1 in the same buffers at pH 3.5–pH 8.0; (C) Ovalbumin incubated alone in solutions buffered in the ranges pH 3.5–pH 8.0, and (D) Ovalbumin incubated with *Fhe*CL1 in the same buffers at pH 3.5–pH 8.0. Digests were analysed by 15% SDS-PAGE. Molecular size markers are indicated on the left.

Since Hb is a major physiological substrate for *Fhe*CL1 we examined the pH dependence for its hydrolysis by *Fhe*CL1 and compared this to the hydrolysis of ovalbumin. Firstly Hb was incubated alone in solutions buffered at various pHs in the range 3.5 to 8.0 for one hour and then analysed by SDS-PAGE. We observed that in the pH range 5.0–8.0 the molecule migrated as a major band at ∼15 kDa representing the Hb-alpha and Hb-beta monomers and a minor band at ∼30 kDa representing the alpha-beta dimers. However, at lower pH values the intensity of the band at ∼30 kDa increased and new bands ≥50 kDa were observed most likely due to aggregation of Hb (with incubation times of greater than one hour precipitation of the Hb was observed in the acidic pH solutions) (Figure 4A).

When Hb (50 µg) was incubated in the presence of *Fhe*CL1 (1 µg) no difference was observed in the migration pattern within the pH range 5.0 to 8.0 (compare Figure 4B with 4A). At pH 4.5, however, addition of *Fhe*CL1 caused the bands at ∼15 kDa and ∼30 kDa to disappear and smearing in the respective lanes indicated the presence of low molecular mass products due to Hb digestion (Figure 4A and B). These Hb bands underwent greater degradation by *Fhe*CL1 in reactions carried out pH 4.0 and 3.5 (Figure 4A and B).

The above results indicate that *Fhe*CL1 cannot digest Hb at pH≥5, whereas digestion is efficient in acidic conditions of pH≤4.5. The lack of digestion at pH≥5 is not due to the inability of *Fhe*CL1 to function in this pH range as the studies above showed that the enzyme could cleave peptide substrates optimally between pH 5.5 and 7.0. To support this observation we analysed the digestion of the protein ovalbumin by *Fhe*CL1 over the pH range 3.5 to 8.0. Ovalbumin incubated in various buffered solutions at pH values from 3.5 to 8.0 migrates in SDS-PAGE as a single band at ∼45 kDa (Figure 4C). When ovalbumin was incubated with *Fhe*CL1 a series of digestive products (<45 kDa) were produced (Figure 4D). SDS-PAGE clearly shows that degradation was optimal between pH 5.0 and 7.0 (Figure 4D), which is in agreement with the optimal activity of the enzyme determined against the peptide substrate (Figure 1C).

### Hb undergoes conformational changes in acidic pH

**Figure 5:**
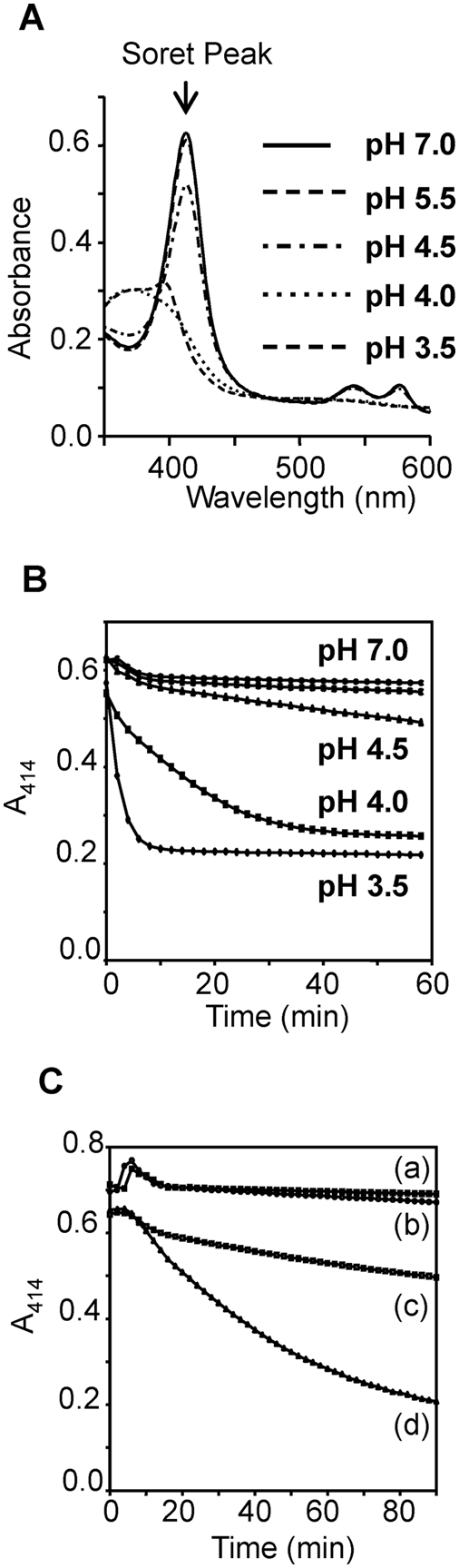
Regulation of *Fhe*CL1 hydrolytic activity against haemoglobin by pH. (A) Spectra of 5.0 µM Hb following 1 hr incubation in 0.1 M buffer at pH 3.5, pH 4.0, pH 5.5 and pH 7.0. Decreases in the Soret peak absorbance at 414 nm shows Hb denaturation with decreasing pH. (B) Progress of denaturation of 5.0 µM haemoglobin at several pH values over time as revealed by the decrease in absorbance at 414 nm. (C) Susceptibility of Hb to *Fhe*CL1 hydrolytic activity at pH 4.0 and pH 7.0 in the presence 1 mM GSH. (a) 5.0 µM Hb and 1 mM GSH at pH 7.0 (b) 5.0 µM Hb, 1 mM GSH and 1 µM *Fhe*CL1 at pH 7.0 (c) 5.0 µM Hb and 1 mM GSH at pH 4.5 (d) 5.0 µM Hb, 1 mM GSH and 1 µM *Fhe*CL1 at pH 4.5.

To investigate the effect of pH on the structure of Hb we obtained absorption spectra of the molecule in various buffered solutions. The absorption spectrum of Hb at physiological pH is characterised by a large Soret peak 414 nm due to the bound heme moiety; disruption of the Hb conformation causes shifts in this peak (Figure 5A). No alteration in the Soret peak was observed between pH 7.0 and pH 5.5, but the height of the peak began to decrease at pH 4.5. When Hb was exposed to pH 4.0 and pH 3.5 the Soret peak completely disappeared (Figure 5A) indicating that structural changes are occurring in the Hb molecule such that it can no longer bind the heme moiety [15].

The denaturation of Hb at low pH was shown to be a time-dependent process (Figure 5B). Progress curves obtained by monitoring the decrease in absorbance at 414 nm clearly show that Hb is stable at pH 7.0 and 5.5 but that partial denaturation occurs at pH 4.5. Hb denaturation was complete at pH 4.0 and 3.5 within one hour.

To determine if the rate of Hb denaturation made it more susceptible to *Fhe*CL1 degradation we mixed Hb at pH 7.0 and pH 4.5 in the presence and absence of 5 µM *Fhe*CL1 and 1 mM GSH and monitored denaturation at 414 nm for 1 hour (Figure 4C). The results show that while *Fhe*CL1 had no effect on Hb denaturation at pH 7.0, the rate of Hb denaturation/digestion was significantly increased at pH 4.5 in the presence of the protease. Thus the Hb molecule at physiological pH is resistant to proteolysis by *Fhe*CL1 but at pH 4.5 alterations in its structure take place that make it susceptible to hydrolysis, which is consistent with our SDS-PAGE analysis described above (Figure 4). Finally, the reducing agent GSH alone, at a concentration of 1 mM, had no effect on Hb denaturation at pH 4.5 or pH 7.0 (Figure 5C).

### *Fhe*CL1 degrades Hb to small peptides but does not release free amino acids

**Figure 6:**
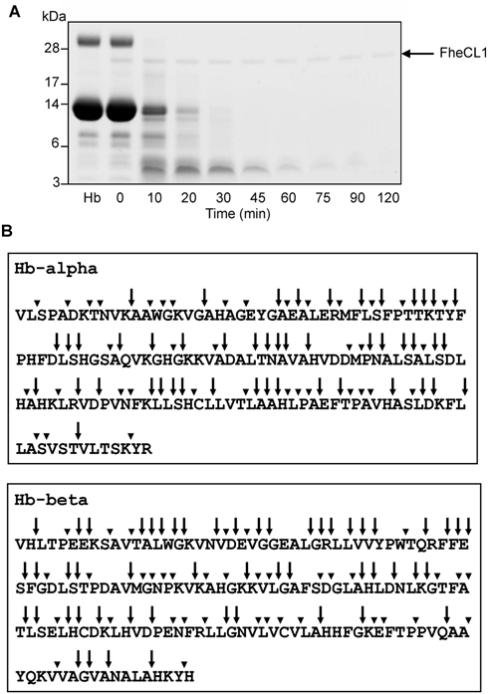
Characterisation of hydrolytic activity of *Fhe*CL1 on Hb. (A) Progress of digestion of Hb by recombinant *Fhe*CL1. Purified haemoglobin (Hb, lane 1) was digested by *Fhe*CL1 in 0.1 M sodium acetate buffer, pH 4.0, containing 1 mM GSH and 1 mM EDTA at 37°C. Reactions were stopped at time 0 and at various time-points (indicated on x axis) by the addition of the cysteine protease inhibitor E-64 and analysed on 4–12% Bis-Tris NuPage gels. The arrow indicates the position of *Fhe*CL1 (25 kDa) that was not degraded in the reaction. Molecular mass markers are shown on the left. (B) Map of Hb α- and β-chains indicating sites of *Fhe*CL1 cleavage the substrates. Cleavage sites within Hb present in 10 min reactions (arrows) compared to cleavages that occur with longer incubation times (120 min, arrowheads) as determined by nanoLc-MS/MS.

To examine the process of Hb degradation by *Fhe*CL1 Hb was mixed with the protease at pH 4.0 for 120 minutes at 37°C. Reactions were stopped at several time points by addition of E-64 (an irreversible inhibitor of cysteine proteases) and the degradation products were analysed by SDS-PAGE (Figure 6A). The bands representing the 15 kDa Hb monomers and 30 kDa Hb dimers were gradually degraded to smaller protein bands in the molecular size region of 3–10 kDa within the first 10–20 minutes of the reaction and completely degraded between 60 and 120 min. It is noteworthy that during this digestive process the *Fhe*CL1 (∼25 kDa) was not degraded (Figure 6A) supporting our earlier data showing that the enzyme is very stable under acid conditions (Figure 2).

**Figure 7:**
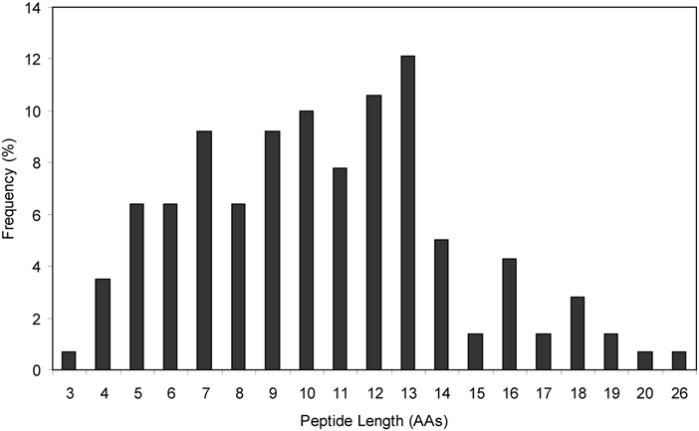
Analysis of peptides released from Hb following digestion by *Fhe*CL1. Frequency (expressed as a percentage) of peptides of varying length released following proteolysis of Hb alpha and beta chains by *Fhe*CL1.

To identify the cleavage sites for *Fhe*CL1 within Hb, the 10 min and 120 min reaction aliquots were analysed by mass spectrometry. The peptides were then mapped onto the primary amino acid sequences of human Hb-alpha and Hb-beta to identify *Fhe*CL1 cleavage sites. Within 10 mins *Fhe*CL1 cleaved Hb-alpha at 47 sites and Hb-beta at 52 sites while at 120 min additional cleavage sites, totalling 83 sites in Hb-alpha and 89 sites in Hb-beta were observed (Figure 6B). Examination of the cleavage map presented in Figure 6B shows that within a 10 min time-frame *Fhe*CL1 could generate small peptides of 4–8 amino acids from Hb. The map also indicates that these would conceivably be further degraded to release dipeptides and free amino acids after 120 mins. The 120 min digests were, therefore, analysed by LC-MS/MS to determine the masses and sequence identities of the resulting hydrolytic products. This analysis revealed that *Fhe*CL1 had degraded Hb into peptides ranging from 3–26 amino acids in length (Figure 7) but not dipeptides or free amino acids. The average length of the released peptides (from both the Hb alpha and beta chains) was 10 amino acids with 13- and 12-residue peptides occurring most frequently in the digested Hb alpha and beta chains, respectively. Accordingly, *Fhe*CL1 must not cleave all Hb molecules in the same manner and, thus, the cleavage map shown in Figure 6B represents a composite of cleavage sites.

To verify that free amino acids and/or small peptides (di- or tri-peptides) were not end-products of the proteolysis the digests were also analysed by MALDI-TOF MS (specifically using the mass range 100 *m/z* to 400 *m/z*). Only 12 mass ions were detected within this range the masses of which five could be mapped to di- or tri-peptides present in either the Hb-alpha or -beta chains. Importantly, ion masses corresponding to free amino acids were not observed.

### *Fhe*CL1 cleavage sites within Hb are consistent with its substrate specificity

**Figure 8:**
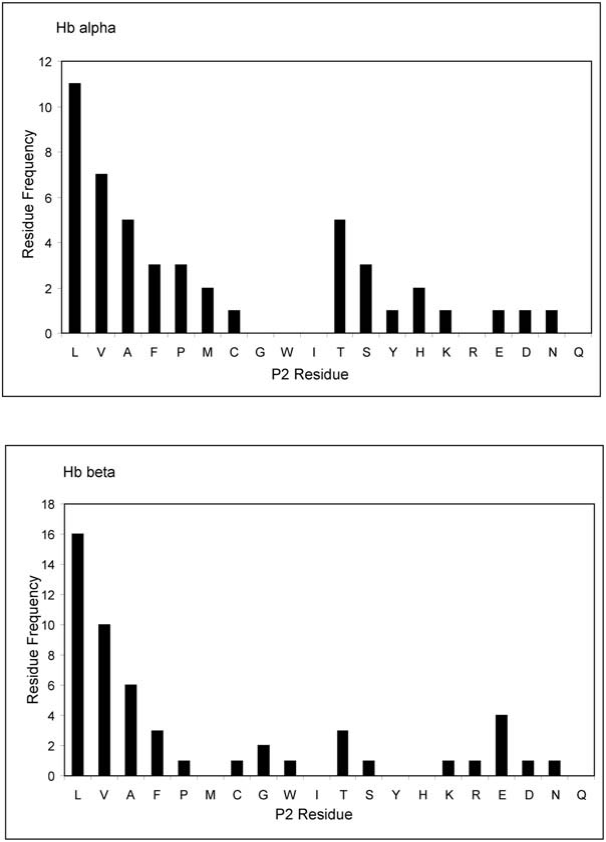
P2 residues in peptides released from Hb following digestion by *Fhe*CL1. Analysis of frequency by which amino acids occur at the P2 position from the peptide bonds cleaved by *Fhe*CL1 in Hb α- and β-chains (corresponding to the 10 min reactions shown in Figure 6). The y axis represents the frequency of a particular residue at the P2 position of the haemoglobin substrates and the x axis shows the amino acids as represented by the one-letter code.

Residues present at the P2 position from the scissile bond interact with the S2 subsite of the active site of papain-like cysteine proteases and determine the efficiency by which the bond is cleaved [21]. Therefore, we examined the frequency of each amino acid in the P2 site of the proteolytic cleavage site identified in aliquots of the 10 min Hb digest described above (Figure 8). Consistent with our previously published studies using fluorogenic peptide substrates and positional-scanning of synthetic combinatorial libraries [13] *Fhe*CL1 preferentially cleaved bonds where the P2 position was occupied with hydrophobic residues; this preference followed the order Leu>Val>Ala>Phe, and was observed for the digestion of both Hb-alpha and Hb-beta (Figure 8). Due to the promiscuity of the *Fhe*CL1 for peptide bonds no obvious trend for P2 preference could be discerned in digests taken at 75–120 minutes (data not shown). Finally, in support of other studies using synthetic combinatorial libraries [13] the P1 position could be occupied by many amino acids but most preferentially Leu.

## Discussion

Proteomic analysis of proteins secreted from adult *F. hepatica* parasites *in situ* within the bile ducts [22] and in culture [22],[23] showed that >80% of the secreted proteins are cathepsin L cysteine proteases. Furthermore, no other class of endopeptidase or exopeptidase was identified in these secretions demonstrating the exclusive reliance of the mature parasites on cathepsin Ls [22],[23]. The cathepsin L proteases are synthesised within epithelial cells lining the parasite gut; these cells have both a secretory and absorptive function and spread extended lamellae into the gut lumen [24]. We have shown that cathepsin L zymogens are concentrated and stored in numerous secretory vesicles that lie at the apex or luminal side of these cells ready for secretion into the gut [7],[24]. By the time the enzymes are secreted outside the parasite they have undergone complete processing to mature enzymes by removal of the prosegment portion, which informed us that the activation process takes place within the gut lumen [12]. The gut lumen of *F. hepatica*, like that of other trematodes such as the schistosomes, is believed to be maintained at a slightly acidic pH, approximately 5.5 [8],[24],[25]. In the present study we have demonstrated using recombinant pro-cathepsin L that auto-catalytic processing and activation can take place at neutral pH but that this occurs far more rapidly at lower pH values (activation at pH 4.0 was 40-times faster than at pH 7.0). Circular dichroism studies showed that the zymogen does not undergo any significant conformational alteration in the pH range 4.0 to 7.0, and like the lysosomal cathepsin Ls of mammals is stable under acid conditions [26]. Enzymatic studies demonstrated that the mature activated enzyme is also very stable under the pH conditions it would experience in the parasite gut. We can conclude, therefore, that the slightly acidic conditions of the parasite gut are very suitable for the autocatalytic activation and digestive function of the cathepsin L proteases.

The primary function of the cathepsin Ls in the parasite gut is to digest host macromolecules and tissues to usable products. Haemoglobin (Hb) is the principle source of amino acids for protein anabolism by the parasite and our present studies demonstrate that *Fhe*CL1 can efficiently degrade this substrate in an acidic environment, pH≤4.5. Surprisingly, however, the cathepsin L protease could not cleave Hb at pHs≥5.0, despite the fact that it has optimal activity towards small-peptide and protein (ovalbumin) substrates between pH 5.5 and pH 7.0. These observations revealed the importance of low pH in regulating the structure of Hb and its susceptibility to proteolysis. By monitoring the Soret peak of Hb over a range of pH values we examined the conformational changes that are induced in the molecule. The Hb molecule retained it structure and bound heme in the pH range 5.5 to 7.0 but partial loss of heme-binding was observed when the pH was reduced to 4.5. Hb underwent full denaturation after 1 hour at pH 3.0–4.0, which in solution could be observed by precipitation of Hb in the reaction tubes. Thus, the susceptibility of Hb to *Fhe*CL1 proteolysis, as revealed by our SDS-PAGE analysis of digestion reactions, correlated with the pH whereby Hb becomes denatured.

A recent study of acid-induced unfolding of Hb monitored by ESI-mass spectrometry proposed the following model for Hb denaturation:
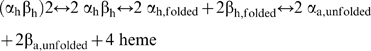
where subscripts “h” and “a” refer to holo- and apo-forms (i.e. heme and non-heme forms of Hb, respectively) [27]. This model indicates that the release of heme from the Hb molecule accompanies the separation and unfolding of the α and β subunits. The final steps in the denaturation scheme, from αβ dimers to heme-bound monomers and then to unfolded non-heme-binding monomers occurred at ∼pH 4.4 and ∼pH 4.0, respectively. In our present study we showed that the addition of *Fhe*CL1 to Hb increased the rate by which Hb lost bound heme at pH 4.5 and confirmed that partial denaturation of Hb at this pH was sufficient to relax the structure of the molecule and make it susceptible to proteolysis. Our results are consistent with a much earlier study by Kimura *et al.* [28] who showed that pH-induced denaturation of Hb increased its susceptibility to trypsin digestion. In conclusion, our studies underscore the importance of the low pH of the parasite gut lumen for denaturing ingested Hb to facilitate its proteolytic hydrolysis. This process is not unlike the denaturation of proteins for hydrolysis in the acid human stomach.

Determining the precise pH of the gut lumen presents a practical hurdle. As mentioned above the pH of the gut lumen in *F. hepatica* has been suggested to be ∼pH 5.5 [8],[25] while that of the related trematode *Schistosoma mansoni* has been estimated to be pH 5.0–6.0 by Senft [29], pH 6.0–6.4 by Chappell and Dresden [30] and 6.84 by Sajid *et al.* [31]. These were not direct measurements of the intraluminal pH but were generally obtained by measuring media into which parasites had extruded their gut contents. Our data showing that *Fhe*CL1 could not digest Hb at pHs≥5.0 is biochemical evidence suggesting that the site of proteolytic activity within the gut must be lower than pH 5.0. Electron micrographs of the gut lumen of both *F. hepatica* [8],[25] and *S. mansoni* [32] often visualise Hb as a dense precipitate, representing presumably denatured protein, in the vicinity of the gut lamellae. Halton's [8],[25] interpretation of micrographs of the gut structure was that digestion in *F. hepatica* takes place between the lamellae of the secretory epithelial cells. Derived from these studies it was suggested that the pH in this local microenvironment is maintained at a more acidic pH than the gut lumen *per se* [24]. In support of this suggestion Delcroix *et al.* [33] found sequestered compartments between lamellae of the schistosome gut with pH as low as 3.9. Their observation explains why the schistosome gut aspartic protease, *Sm*CD, whose activity is confined to the range of pH 2.5–4.6 [34] could participate in Hb digestion. Similarly it explains the role of the schistosome cathepsin L cysteine protease, *Sm*CL1, which could efficiently cleave Hb only in the pH range 4.0–4.5 [14].

*Fhe*CL1 and other papain-like cysteine proteases are activated in the presence of low molecular mass thiols such as cysteine or DTT [35],[36]. Although these compounds are routinely used to activate cysteine proteases as part of *in vitro* activity assays, they are not considered physiologically relevant reducing agents. GSH is the most abundant intracellular reducing agent and its concentration inside red blood cells is particularly high, estimated to be 1.192 mM by Mills and Lang [20] and ∼3.2 mM by Chappell *et al.* [37]. Here we found that GSH effectively enhances *Fhe*CL1 activity towards small synthetic substrates with an optimum at ∼0.1 to 1.0 mM GSH, and accelerates the digestion of Hb by *Fhe*CL1. A concentration of ∼0.1 to 1.0 mM GSH could conceivably be reached in the parasite gut following lysis of ingested red blood cells notwithstanding variations in the size of the blood meal and dilution in the parasite gut.

We used mass spectrometry to identify the cleavage sites of *Fhe*CL1 within Hb and to determine the size of the peptide products generated by its complete digestion. *Fhe*CL1 digested Hb at 83 cleavage sites in Hb-alpha and 89 sites in Hb-beta that resulted in short peptides of at least 4–14 amino acids, with some appearance of tripeptides. Residues in the P2 position are known to influence the efficiency of all papain-like cysteine proteases, and we found that those residues in Hb that were most susceptible to cleavage by *Fhe*CL1 were invariably a hydrophobic residue, and in the order Leu>Val>Ala>Phe. These results are consistent with our earlier studies using fluorogenic peptides and peptide libraries that showed *Fhe*CL1 to have a more restricted S2 active site compared to human cathepsin L and most readily accommodates hydrophobic P2 residues, particularly Leu [13]. It is pertinent to note that the amino acids Leu, Val, Ala, Phe make up approximately 42% of the Hb molecule and, therefore, we would propose that *Fhe*CL1 has been specificity adapted to degrade this substrate. However, our studies also show that cleavage by *Fhe*CL1 does not generate free amino acids and, by extension, suggests that Hb degradation is not completed within the parasite gut but that small peptides are taken up by the gut epithelial cells during their absorptive phase for further processing within cells [24]. The enzymes involved in this process likely include a dipeptidylpeptidase [10] and an aminopeptidase [11] that function at neutral pH and have been located by immunofluorescence microscopy within the cytosol of the epithelial cells.

Our studies on *F. hepatica* point to a digestive machinery that requires proteases of only one mechanistic class i.e. cathepsin cysteine proteases. However, *F. hepatica* secretes different forms of these proteases with overlapping specificity that may complement each other [23]. Nevertheless, the mechanism of gut digestion appears to differ markedly from other helminths so far studied. Dalton *et al.* [38] were first to propose that schistosomes exploit a cascade involving aspartic and cysteine (cathepsin B, L1 and L2) proteases within their gut lumen to achieve the complete degradation of Hb. The more recent studies by Delcroix *et al.* [33], which exploited selective protease inhibitors and RNA interference (RNAi) to explore the mechanism of Hb digestion in schistosomes, supports the role of a network or combination of cysteine proteases, aspartic protease and an asparaginyl endopeptidase. However, Correnti *et al.* [39] showed that while knockdown of cathepsin B expression in schistosomes by RNAi retarded parasite growth it did not prevent Hb digestion in the parasite gut. On the other hand, Morales *et al.* [40], also using RNAi, demonstrated that the cathepsin D aspartic protease is essential to survival of schistosomes through its pivotal role in Hb digestion.

A multi-enzyme cascade involving cysteine and aspartic proteases is also necessary for Hb digestion in canine hookworm *Ancylostoma caninum* [41] and aspartic and cysteine proteases in the nematode *Ostertagia ostertagi* have also been shown to have activity against Hb [42]. Although several proteases appear to be involved in Hb digestion in these helminths it is still not clear whether digestion is regulated in an ordered manner, each enzyme working sequentially, or whether all proteases work simultaneously and in a random manner. Dalton *et al.* [7],[24] suggested that the activity of each protease within the gut was regulated by pH, and therefore as the bloodmeal (pH 7.0) was drawn into the gut the pH slowly decreased (perhaps by proton pumps in the epithelial cells), each enzyme would come into play when its appropriate pH range for activity was reached; thus in schistosomes cathepsin B (optimum pH 4.0–6.0) would be activated before cathepsin L (optimum pH 4.0–4.5), which would be followed by aspartic proteases (optimum pH 2.9–4.0).

The cathepsin L proteases of *F. hepatica* also participate in functions outside the parasite gut; these include liver tissue degradation, cleavage of host antibodies and suppression of host immune cell function (see [6]). The blind-ended gut of the parasite is emptied every 3 hours, thus depositing the cathepsin L proteases outside [9]. The extracorporeal roles of the proteases are performed at pH values that are between two and three pH units higher than the microenvironment at which the proteases function in the parasite gut. Our studies showing that *Fhe*CL1 are active and highly stable at neutral pH points to a specific adaptation of these molecules to carry out functions over a wide pH range. It is interesting to note that the pH optimum of the *Fhe*CL1, pH 6.2, is approximately mid-point between the pH values at which it works inside and outside the parasite. In contrast, lysosomal cathepsin Ls of mammals are active only at pHs values of approximately 4.5, in keeping with the environment in which they function, and are inherently unstable at neutral pH so that cellular damage due to leakage from the lysosome is avoided [43].

To conclude, the helminth parasite *F. hepatica* secretes cathepsin L proteases that are specifically adapted to be functional at pHs at which they perform essential roles in this parasite's biology. The low pH of the parasite gut is important in regulating the activity of these proteases by providing a milieu whereby the proteases readily autocatalytically activate from inactive zymogens secreted by the surrounding epithelial cells, and by facilitating the denaturation of the protein substrates on which the proteases act. The mature cathepsin L proteases are extremely stable at this pH and their hydrolytic activity is greatly enhanced by GSH, most likely derived from ingested host red blood cells. *Fhe*CL1 is specifically designed to cleave peptide bonds with N-terminal hydrophobic residues which are most common in Hb with the goal to provide small peptides that can be absorbed by the gut epithelial cells for further processing to amino acids within cells before distribution to parasite tissues *via* amino acid transporters [24]. However, following completion of the digestive process in the gut lumen unwanted material is extruded which delivers the proteases to the outside where they can perform their additional extracorporeal roles at physiological pH conditions in which they are also highly active and stable.
